# Osteoid Osteoma Mimicking Triangular Fibrocartilage Complex Injury: Diagnosis and Review of Treatment

**DOI:** 10.1155/2012/612106

**Published:** 2012-09-10

**Authors:** J. M. Lamo-Espinosa, A. González, S. Amillo

**Affiliations:** University Clinic of Navarra, C/Pío XII 36, Navarra, 31008 Pamplona, Spain

## Abstract

We report the case of osteoid osteoma (OO) with ulnar styloid involvement. A review of the literature has been made with the aim of defining the special behaviour of OO when it is near the articular surface. That behaviour can affect the diagnosis, masking the real etiology of the pain, delaying the diagnosis, missing the diagnosis, or what is more serious, conducting an inadequate treatment. We propose a treatment algorithm conducted based on the localization near or far from articular surface and the superficial or deep localization.

## 1. Introduction

Bergstrand made the first description of osteoid osteoma (OO) in 1930 and Jaffe described it as an independent identity in 1935.

The OO accounts for 4% of all primary bone tumours with a male : female ratio of 2 : 1 being most frequently diagnosed in the second decade of life.

Any bone can be affected by OO. The proximal femur and tibia are the most often involved, being the location in the hand and wrist rare [1]. Some studies suggest a frequency of upper extremity of 19% to 31%.

The etiology of OO has not been yet clarified. Histologically we highlight a nidus, with high amount of prostaglandins within highly vascularized, surrounded by a thin but dense layer of reactive bone [1].

Pain is the first symptom of this tumour, which subsides with most NSAID, explained perhaps by the amount of prostaglandins containing in the nidus.

## 2. Clinical Case

A 37-years-old right-handed man with pain in his right wrist of 3 years evolution, signed a fingertip on the ulnar side, constant with inflammatory characteristics, which wakes him up at night. He does not associate pain with any movement of the wrist although it calms temporarily with NSAIDs. He correlated the onset of pain with an overload made at the same time of the onset of pain. 

Diagnosed in another center of triangular fibrocartilage complex injury, he was operated one year ago with arthroscopic technique without improvement of symptoms. 

Physical examination revealed an articular balance preserved with a wrist flexion of 88°, an extension of 70°, 45° of ulnar deviation, 25° of radial deviation, and a pronation-supination movement of 90°. He had no trophic changes and shows intense pain with the palpation of right ulnar styloid. The force was preserved with a normal neurovascular examination.

**Figure 1:**
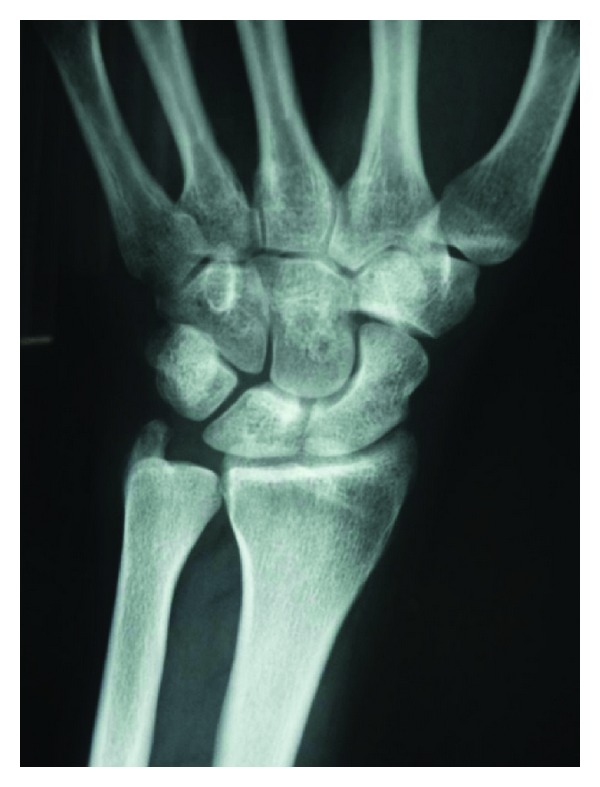
Visualization of lytic lesion of 0.4 mm diameter with a sclerotic line surrounding the ulnar styloid apophyses.

**Figure 2:**
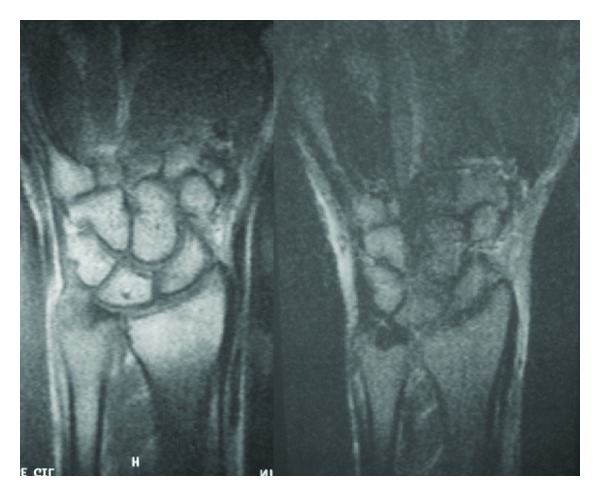
Wrist magnetic resonance scan where the T2-weighted sequence revealed a high-hyperintense signal in area of the triangular fibrocartilage and synovium. It did not demonstrate the characteristic nidus of osteoid osteoma.

Anteroposterior and lateral wrist radiographs are performed objectifying a lytic lesion of 0.4 mm diameter with sclerotic line surrounding in the ulnar styloid apophyses (Figure 1). The patient provided a wrist arthroresonance where in T2-weighted sequence revealed a high-hyperintense signal in the area of the triangular fibrocartilage and synovial. It wasn not an appreciated characteristic of osteoid osteoma's nidus (Figure 2).

**Figure 3:**
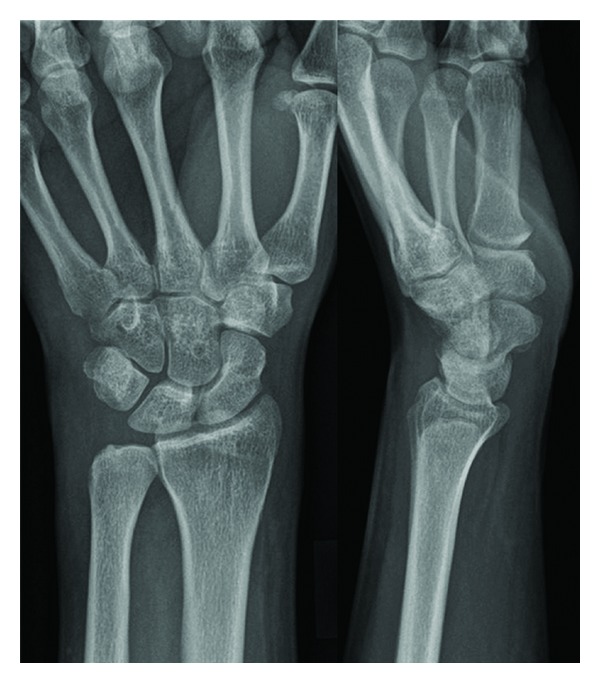
An open surgical treatment was performed, with resection of the styloid process of ulna, synovectomy, and inspection of the review fibrocartilage triangular, which was intact. The material was sent to pathology, reported as osteoid osteoma with involvement of the synovial tissue with chronic fibrovascular changes.

An open surgical treatment was performed, with resection of the styloid process of ulna associating synovectomy with review of the fibrocartilage triangular, which was intact (Figure 3). The material was sent to pathology anatomy study, reported as OO with involvement of the synovial tissue with chronic fibrovascular changes.

The patient progressed well, with disappearance of pain immediately after surgery. Revaluated at 6 weeks after operartion he had full wrist mobility without signs of recurrence one year after procedure.

## 3. Discussion

Numerous studies report wrist's localization of OO. Murray et al. [2] identified 44 carpal's tumours of total of 26800 bone tumours, constituting a prevalence of 0.04%. The large case series referred to the upper extremity is published by Bednar et al. [1]. He found 46 patients with OO with only 2 distal ulna localization, not referring specifically styloid involvement. At 2002 Marcuzzi et al., in their study of 18 bone tumours, did not find any ulnar involvement.

On the other hand, affectation of radial styloid has been described in the literature [3]. The peculiarity of this localization is that clinically mimics symptoms of De Quervain tendinitis analogous with our findings on the ulnar side that mimics a tenosynovitis' and fibrocartilages' lesion. That is why it was initially treated looking for a triangular fibrocartilage rupture.

Inflammatory changes in the wrist, which can be created by juxtaarticular OO, were already demonstrated by Sherman [4] after analysing the arthritic changes and proliferative response secondary to synovial inflammatory stimulus caused by osteoid osteoma. Lymphoproliferative synovial, similar to that found in patients with arthritis was found by Snarr et al. [5], making synovial biopsy in patients with osteoid osteoma next to the joint but in our opinion clinically, these changes are different from those produced in arthritis.

Therefore, delayed diagnosis and inadequate treatment of OO is greater in cases in the proximity to the joint. In 2 studies with large number of cases involvement the hand and wrist, the time elapsed since the onset of symptoms to definitive treatment was 14 and 16 months. In our case it was 36 months.

Approximately 13% of cases occur in patients over 30 years, not being the age factor of exclusion diagnosis. Our patient is 37 years at diagnosis.

Although it has been reported that some limitation in mobility by secondary changes in the synovium or the disruption of the cortex occur, in our case the patient had no limitation.

The diagnosis of OO was carried out with the detailed observation of the anteroposterior and lateral radiographs of the wrist. Swee et al. reports that up to 25% of cases are not demonstrable on plain radiography, getting the diagnosis with the CT-imaging technique that is most often used. Some authors reported in the literature negative results with this technique. Pin et al. recommended scintography as a more sensitive technique for detecting focal lesions in patients with chronic inflammatory pain in the wrist of unknown cause. In our case no more studies were necessary.

In recent years the treatment of OO has been the subject of numerous studies on orthopaedics surgery because the use of radiofrequency with or without CT guided.

There are studies that propose the use of NSAID for the treatment of osteoid OO. In 1992 Kneins et al. referred to control symptoms of 9 patients treated with NSAID, achieving a resolution of symptoms definitively in 6 of them at a median of 33 months which coincides with the study of Ilyas and Younge et al. with results of resolution in 6 of the 11 patients. All of these are poorer results than surgical treatment.

Open surgery has been the gold standard treatment for OO, with rates of complete remission without recurrence of pain from 88% to 100%. Open surgery involves bloc resection, the curettage filled with cancellous allograft bone, or structural allograft. Supporters of the radiofrequency as definitive treatment often used as a reference Rosenthal's study [6], where comparing the use of open surgery with radiofrequency. In his results shown, in those patients in whom open surgery, a hospital stay of 4.7 days without full weight bearing more than 3 months associated with limitation of usual activity during this time. Soong et al. reports a resolution of pain 76% of patients with radioablation in the upper extremity, lower than in studies related to open surgery [7].

Among the disadvantages that have been attributed to open surgery treatment are prolonged anaesthesia time, the need for greater exposure with the secondary damage of soft tissues, and the longer hospital stay. Akhlaghpoor et al. [8] published a series of cases treated with radiofrequency. Although they report good results we find the limitations derivated of no pathologic anatomy diagnosis (considering the final diagnosis obtained by the imaging and clinical of the patient) and the limitation secondary to the lost follow up long term. We must note the recurrences described on the literature were until 44 months of alter ablation.

The percutaneous CT-guided excision is a minimally noninvasive procedure with success rates of 77% to 100% with the advantage of obtain a biopsy to confirm the diagnosis. The problem of the technique, is that it cannot be used in superficial localizations and closed to the joint surfaces [9] because the risk of burn injury (if it is associated a radiofrequency ablation) and the impossibility to reconstruct with allograft. The average duration of this procedure is 1.25 to 4 hours. Similar time was spent in our case.

In our case, because the superficial location of the lesion we decided to perform an open resection without the disadvantages described in the literature for open surgery and without the morbidity associated with the 2 percutaneous treatments. With a direct approach to the ulnar styloid with 3 cm incision and local anaesthesia and hospital stay of 1 day alter it with no morbidity associated with use of graft that was not necessary. We have the advantage of obtaining a pathological diagnosis of the injury, one of the limitations of using radiofrequency ablation alone. Without radiofrequency ablation we prevent possible skins or joints complications.

Resolution of pain in our case occurs in the first 24 hours, coinciding with the study by Ilyas and Younge [10] with rates of 100% resolution at 48 hours (91% at 24 hours).

Case series of patients treated with radioablation show complete resolution of symptoms between 1 and 2 weeks after ablation.

Although is rare, osteoid OO should be considered in the differential diagnosis of chronic wrist pain. The inflammatory response of the synovium of unknown origin may mask the presence of bone injury.

**Figure 4:**
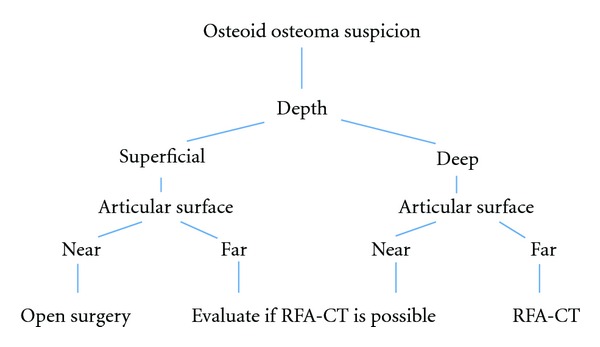
Algorithm propose of treatment.

The treatment of OO should be individualized as a function of localization (depth and articular proximity) (Figure 4) as well as the need for confirmation of diagnosis with biopsy, recommending open surgery on superficial cases or closed to the articular surface, and CT-guided radiofrequency in depth without articular involvement. We do not recommend the treatment with radiofrequency ablation alone.
